# Novel synthetic cyclic integrin αvβ3 binding peptide ALOS4: antitumor activity in mouse melanoma models

**DOI:** 10.18632/oncotarget.11363

**Published:** 2016-08-18

**Authors:** Shiri Yacobovich, Lena Tuchinsky, Michael Kirby, Tetiana Kardash, Oryan Agranyoni, Elimelech Nesher, Boris Redko, Gary Gellerman, Dror Tobi, Katerina Gurova, Igor Koman, Osnat Ashur Fabian, Albert Pinhasov

**Affiliations:** ^1^ Department of Molecular Biology, Ariel University, Ariel, Israel; ^2^ Department of Cell Stress Biology, Roswell Park Cancer Institute, Buffalo, New York, USA; ^3^ Department of Chemical Sciences, Ariel University, Ariel, Israel; ^4^ Department of Human Molecular Genetics and Biochemistry, Sackler School of Medicine, Tel Aviv University, Tel Aviv, Israel

**Keywords:** cancer, cyclic peptide, integrin, ALOS4, melanoma

## Abstract

ALOS4, a unique synthetic cyclic peptide without resemblance to known integrin ligand sequences, was discovered through repeated biopanning with pIII phage expressing a disulfide-constrained nonapeptide library. Binding assays using a FITC-labeled analogue demonstrated selective binding to immobilized αvβ3 and a lack of significant binding to other common proteins, such as bovine serum albumin and collagen. In B16F10 cell cultures, ALOS4 treatment at 72 h inhibited cell migration (30%) and adhesion (up to 67%). Immunofluorescent imaging an ALOS4-FITC analogue with B16F10 cells demonstrated rapid cell surface binding, and uptake and localization in the cytoplasm. Daily injections of ALOS4 (0.1, 0.3 or 0.5 mg/kg i.p.) to mice inoculated with B16F10 mouse melanoma cells in two different cancer models, metastatic and subcutaneous tumor, resulted in reduction of lung tumor count (metastatic) and tumor mass (subcutaneous) and increased survival of animals monitored to 45 and 60 days, respectively. Examination of cellular activity indicated that ALOS4 produces inhibition of cell migration and adhesion in a concentration-dependent manner. Collectively, these results suggest that ALOS4 is a structurally-unique selective αvβ3 integrin ligand with potential anti-metastatic activity.

## INTRODUCTION

Many cancers have common, overexpressed molecular elements associated with the diseased state and include several membrane proteins, such as cytokine/chemokine receptors, receptor tyrosine kinases, and integrins [1]. Integrins are heterodimeric cell-surface glycoproteins that trigger a diversity of signaling pathways controlling cell adhesion, proliferation, survival, morphology, motility, cell cycle progression and cell differentiation [2, 3]. They are critical for angiogenesis [1], lymphangiogenesis [1], thrombocytosis [4, 5] and immune response [6–8] and are involved in various human pathologies including inflammation [9, 10], fibrosis [9], infectious diseases [11–13] and cancer [1]. Integrins, particularly αvβ3, are involved in angiogenesis through interaction with vascular epithelial growth factor (VEGF) receptor, fibroblast growth factor 2 (FGF2), platelet-derived growth factor (PDGF), and matrix metalloproteinase-2 (MMP-2) which collectively aid tumor development and growth [14, 15]. It has been hypothesized that selective integrin expression allows tumor cells to metastasize to different organs [16–18], thus integrins represent an attractive target for the prevention of cancer spread and tumor progression.

Inhibition of integrin αvβ3 as a cancer treatment could be potentially effective since its expression is highly upregulated in different malignancies, including melanoma [19], glioma [20], prostate carcinoma [21–23], bone metastasis [23, 24] and breast cancer [17]. A common integrin binding motif, Arg-Gly-Asp (RGD), is shared by several extracellular matrix (ECM) molecules, including fibronectin, vitronectin, and fibrinogen [25]. There have been attempts to develop inhibitors of αvβ3 interactions with matrix protein and some of them, such as cyclic peptide (Cilengitide [26]) and functional anti-αvβ3 antibodies (Abegrin [27]), show anti-cancer activity *in vitro* and *in vivo*. However, recent studies demonstrate that αvβ3 also binds proteins that do not contain canonical RGD sequences [28].

In the present study, based on a screened phage library displaying disulfide-constrained nonapeptides against integrin αvβ3, we identified a novel non-RGD peptide with high binding affinity to αvβ3. This peptide demonstrated strong antitumor activity in mouse models of local and metastatic melanoma.

## RESULTS

### Phage selected by αvβ3 integrin binding displays non-RGD motifs

To identify cyclic peptides that bind to the integrin αvβ3, an M13 phage peptide library was used with inserts coding cysteine-flanked random heptapeptide (forming nonapeptides). A disulfide bond formed by these cysteines cyclized the sequences, resulting in libraries expressing conformationally-constrained peptides allowing high-affinity binding with receptors. The screening procedure was repeated twice and total of 5 DNA sequences with integrin binding affinity were identified. Peptide with amino acid sequence CSSAGSLFC (MW = 871.98) and corresponding nucleotide sequence TGT TCT TCT GCT GGT TCT CTT TTT TGC was discovered and named ALOS4. Bioinformatic analysis did not reveal any sequence homology of ALOS4 to known integrin binding ligands [29].

### Selective binding of ALOS4 to integrin αvβ3

**Figure 1:**
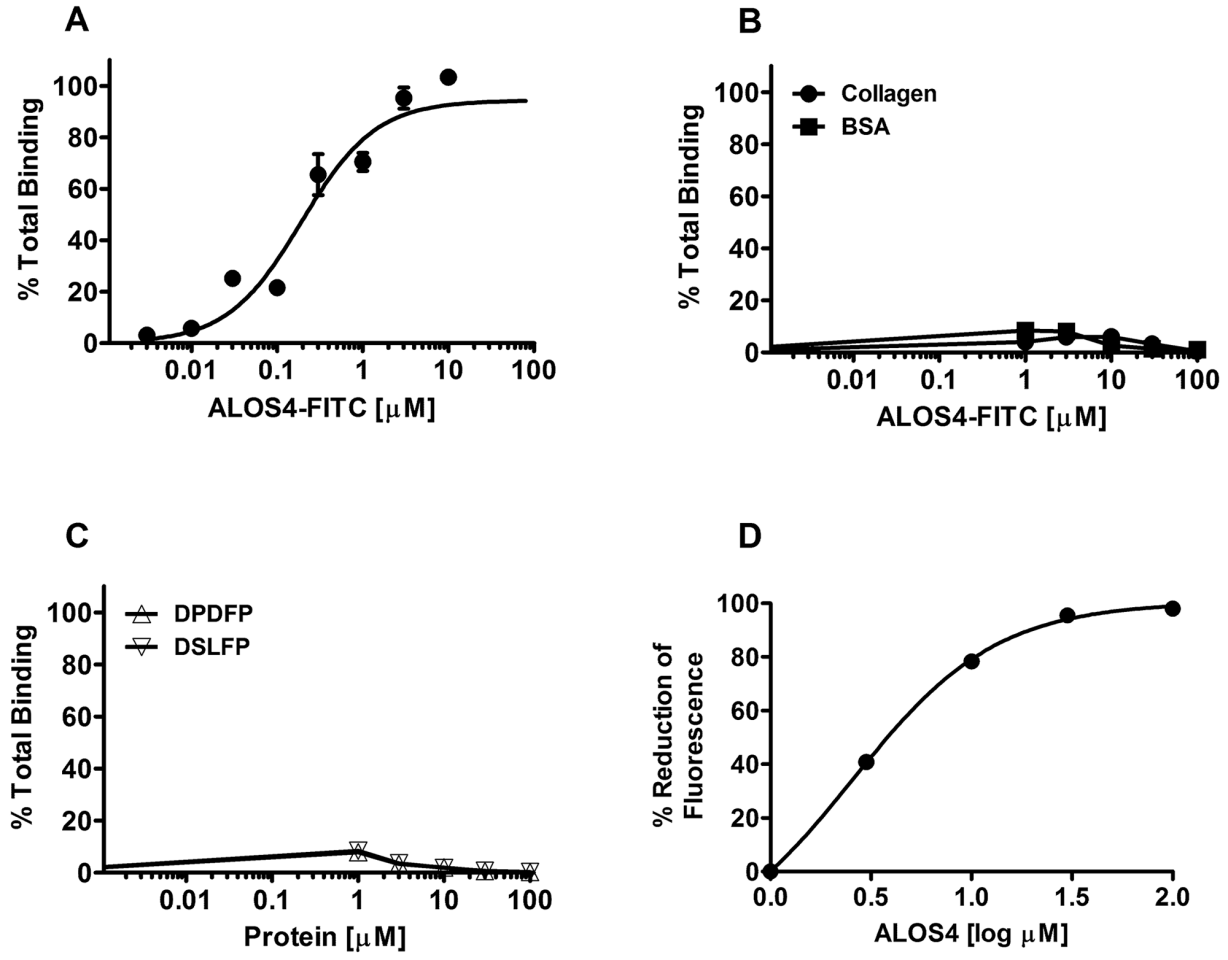
Selective binding of ALOS4 to αvβ3 **A.** ALOS4-FITC binding in wells pre-coated with integrin αvβ3. ALOS4 binding to αvβ3: Kd = 0.192±0.038 μM. **B.** ALOS4-FITC binding in wells coated with collagen or BSA. **C.** Binding of random, FTIC-labeled cyclic nonapeptide controls (DPDFP and DSLFP) in wells pre-coated with integrin αvβ3. **D.** Competitive homologous binding of ALOS4 to αvβ3 integrin. 10 μM of ALOS4-FITC peptide was incubated with different concentrations of unlabeled ALOS4 on wells pre-coated with αvβ3. Kd = 2.55±1.22 μM. Data are expressed as percent reduction of maximal fluorescence signal.

Binding affinity of ALOS4 to immobilized purified αvβ3 was evaluated using saturation and heterologous competition assays. Fluorescence intensity was positively correlated with concentration of FITC-labeled ALOS4 peptide (FITC-ε-Acp-CSSAGSLFC; MW=1374.54) when tested on wells coated with αvβ3 (estimated Kd=0.192±0.038 μM; Figure 1A). ALOS4 displayed no affinity for bovine serum albumin or collagen (Figure 1B). Two labeled control random cyclic nonapeptides (DFDFP and DSLFP) also did not display any significant binding to αvβ3 (Figure 1C). By employing heterologous competition binding experiments between FITC-labeled ALOS4 and non-labeled ALOS4, we found that fluorescence intensity reduction was correlated with concentration of unlabeled ALOS4 cyclic peptide with estimated Kd=2.55±1.22 μM (Figure 1D).

### ALOS4 does not reduce cancer cell viability, but has an effect on cell migration and adhesion *in vitro*

**Figure 2:**
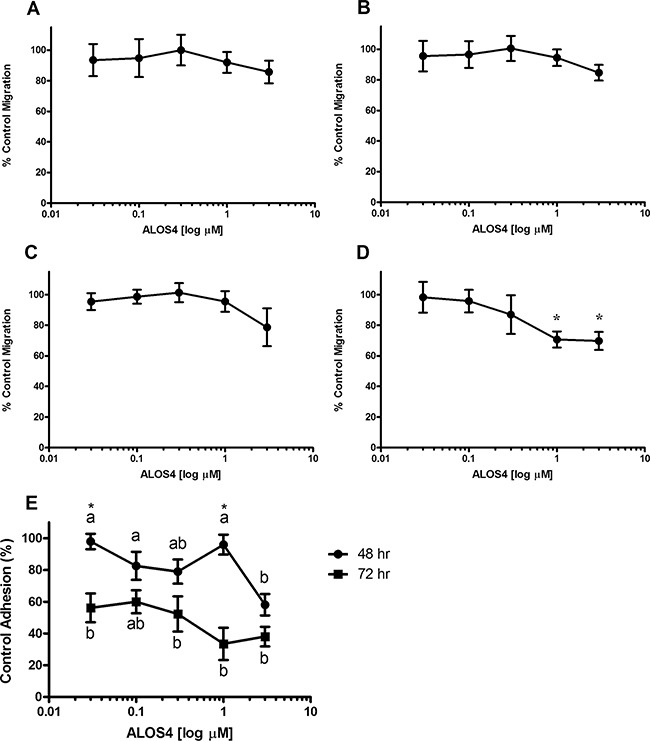
ALOS4 reduces cell migration and adhesion Percent control wound closure at 8 h in B16F10 cells treated for **A.** 24, **B.** 48, **C.** 60 and **D.** 72 h with ALOS4 (one-way ANOVA followed by Bonferroni means separation test; p=0.0242, *p<0.05, n=3). **E.** Cells were pretreated for 24 or 48 h with concentrations of ALOS4 (0.03-3 μM), then replated at a fixed cell concentration per well with an additional 24 h of ALOS4 treatment (n=4). Letters are results of a one-way ANOVA within treatment with Bonferroni means separation test (48 h: p=0.0107; 72 h: p=0.0005). Asterisks are results of a two-way ANOVA comparing pretreatment times (p=0.0072; *p<0.05).

Since the role of integrins has been demonstrated in melanoma growth and progression [19, 30], we conducted a cursory investigation of potential mechanisms of action of ALOS4 on B16F10 mouse melanoma migration, adhesion and viability. Results suggested a small concentration-dependent inhibition of cell migration up to 60 h of ALOS4 treatment, however the effect was not significant (Figures 2A-C). We did observe, however, that ALOS4 inhibited cell migration (30%) at concentrations as low as 1 μM (p<0.05) following 72 h of drug exposure (Figure 2D). Treatment of B16F10 cells with ALOS4 (0.03-3 μM) at 3 μM following 48 h of treatment produced 42% inhibition of cell adhesion (Figure 2E). Longer treatment times (72 h) resulted in 44-67% inhibition of cell adhesion at concentrations as low at 0.03 μM, which appeared to follow a concentration-dependent trend. However, we did not observe an effect of ALOS4 at a range of concentrations (0.005-100 μM) on B16F10 cell viability *in vitro* using resazurin assay (data not shown).

### Cellular binding and translocation of ALOS4 from cell surface to cytoplasm proceeds rapidly after exposure

**Figure 3:**
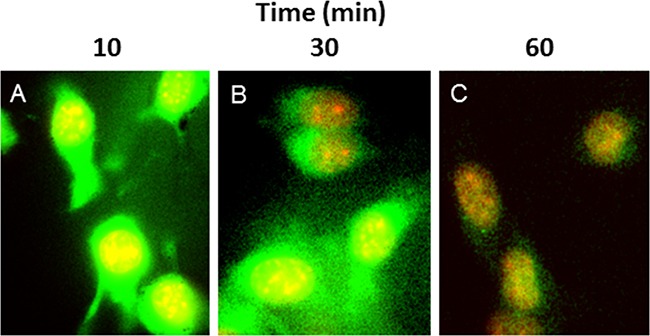
ALOS4 readily enters the cytoplasm of cells and gradually migrates to the nuclear envelope B16F10 cells incubated with ALOS4-FITC (green label) and DAPI (red label) at **A.** 10 min, **B.** 30 min and **C.** 60 min ALOS4-FITC exposure. Yellow indicates sites of colocalization.

Immunofluorescence microscopic examination of ALOS4-FITC association with B16F10 cell membranes indicated that the peptide rapidly adheres to the cell surface (by 10 min), then appears to translocate quickly to the cell cytoplasm (30 min; Figure 3). Additional incubation times up to 60 min indicated that a majority of ALOS4-FITC migrated to the nuclear envelope and/or surrounding endoplasmic reticular membranes, leaving very little peptide in the cytoplasm. Additional studies are currently underway to better characterize this phenomenon using an ALOS4- and integrin-specific monoclonal antibodies.

### ALOS4 inhibited growth of local and metastatic cancer in mouse model of aggressive melanoma

Although we did not see an effect of ALOS4 on growth of tumor cells *in vitro*, inhibition of cell migration and adhesion by ALOS4 suggested that it has inhibitory activity on integrin signaling, which is vital for the growth of tumors *in vivo*. Since the role of integrins in melanoma has been demonstrated in multiple studies [19, 30–33], we therefore decided to examine anti-tumor activity of ALOS4 in mouse model of B16F10 melanoma.

**Figure 4:**
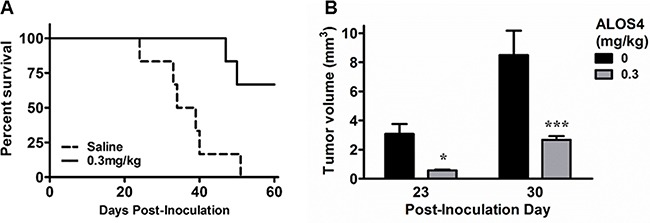
ALOS4 dose-dependently increases survival of mice and reduces tumor mass in subcutaneous melanoma model Mice (16 week-old, C57BL/6; n=10 each group) were inoculated s.c. with B16F10 cells (5.0 × 10^4^ cells/ml) and injected daily with ALOS4 (0.1, 0.3 or 0.5 mg/kg, i.p.) from day 1-60 post-inoculation. Results were similar for all ALOS4 doses examined; only 0.3 mg/kg ALOS4 shown for clarity. **A.** ALOS4-treated mice had significantly higher survival (Mantel-Cox, P<0.0001; Log-rank test for trend, p=0.0016). Median survival date of animals: Saline control, 36.5 days; ALOS4 0.3 mg/kg-treated, could not be estimated. **B.** Palpable tumor mass was estimated by (π/6 × width × length × height). Bars represent mean tumor volume (±SEM). Asterisks represent results of a Bonferroni means separation test between doses (two-way ANOVA, p=0.0002; *, p<0.05; ***, p<0.001).

First we tested if ALOS4 would inhibit melanomas cell engraftment. We subcutaneously inoculated B16F10 cells into mouse flanks and started ALOS4 administration 24 hours after inoculation. ALOS4 daily treatments (0.3 mg/kg i.p.) significantly increased survival in comparison with saline-treated controls (Mantel-Cox, p<0.0001; Log-rank test for trend, p=0.0016; Figure 4A). Thus ALOS4 has anti-tumor effect *in vivo*.

Next we sought to determine if ALOS4 would inhibit growth of established tumors. Mice flank-inoculated s.c. with B16F10 cells were treated with ALOS4 or saline one day after inoculation. Twenty three days after start of peptide administration, ALOS4-treated mice had significantly smaller tumors than control animals (Saline-treated: day 23, 3.08±0.69 mm^3^; day 30, 8.49±1.68 mm^3^. ALOS4-treated [0.3 mg/kg]: day 23, 0.57±0.06 mm^3^; day 30, 2.68±0.25 mm^3^; two-way ANOVA, p=0.0002; Figure 4B).

**Figure 5:**
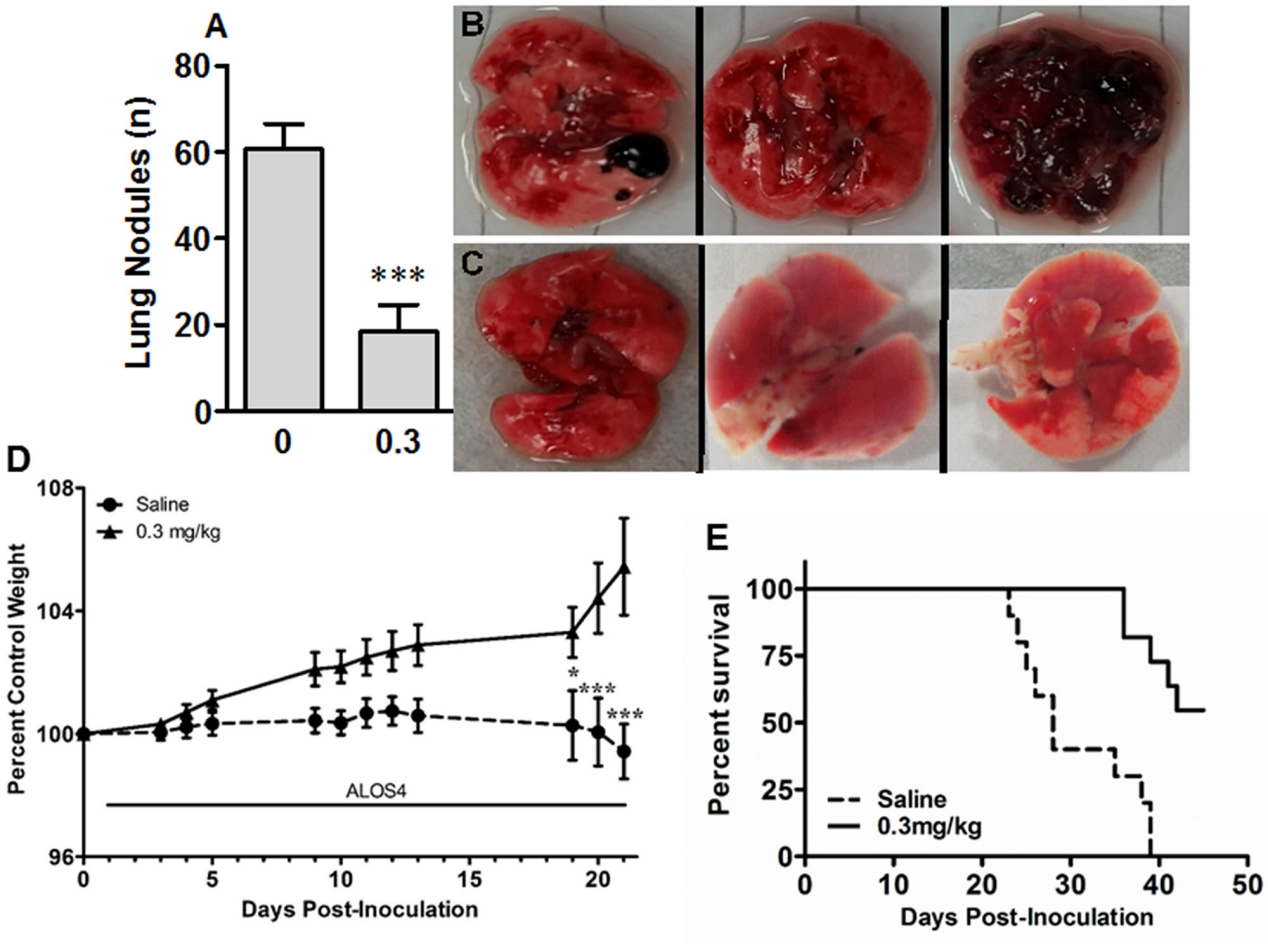
ALOS4 reduces cancer nodule formation in lungs, reduces weight loss and increases survival in metastatic melanoma mouse model Mice (16 week-old, C57BL/6; n=6 each group) were inoculated with B16F10 cells (5.0 × 10^4^ cells/ml) and injected daily with ALOS4 (0.3 mg/kg, i.p.) from day 1-18 post-inoculation. **A.** Lung nodule counts from gross anatomical inspection of lung tissue from saline-injected and 0.3 mg/kg ALOS4-injected mice (ALOS4-treated, n=6, saline-treated, n=7; ***, p<0.001). Bouin's solution-fixed example lungs from **B.** saline-injected and **C.** 0.3 mg/kg ALOS4-injected mice. **D.** Mice (16 week-old, C57BL/6) were inoculated with B16F10 cells (5.0 × 10^4^ cells/ml) and injected daily with ALOS4 (0.3 mg/kg, i.p.) from day 1-21 post-inoculation. Weights were determined at least 3x/week. Data are expressed as percent of control (starting) weight (n=6 mice per group). Asterisks are results of a Bonferroni means separation test (Two-way ANOVA, p<0.0001; *, p<0.05; ***, p<0.001). **E.** Mice (16 week-old, C57BL/6; n=6 each group) were inoculated with B16F10 cells (5.0 × 10^4^ cells/ml) and injected daily with ALOS4 (0.1, 0.3 or 0.5 mg/kg, i.p.) from day 1-45 post-inoculation. Results were similar for all ALOS4 doses examined; only 0.3 mg/kg ALOS4 shown for clarity. ALOS4-treated mice had significantly increased survival (Mantel-Cox, p<0.0001; Log-rank test for trend, p=0.0004). Median survival date of animals: Saline control, 28 days; ALOS4 0.1, 0.3, 0.5 mg/kg-treated, could not be estimated.

Finally we assessed the effect of ALOS4 on the growth of lung metastases established via tail vein inoculation of B16F10 melanoma cells. Daily ALOS4 administration at 0.3 mg/kg one day after tumor cell inoculation significantly reduced the number of metastases in the lungs (Saline-treated: 60.6±6.0; 0.3 mg/kg ALOS4-treated: 18.3±6.3; unpaired t-test, p<0.001; Figure 5A-C). Moreover, saline-treated animals started losing weight from day 19, whereas ALOS4-treated mice gained weight during the entire experiment period (percent control weight for post-inoculation days 19, 20 and 21, respectively: Saline-treated: 100.27±1.13, 100.06±1.10, 99.42±0.98; 0.3 mg/kg ALOS4-treated: 103.30±0.82, 104.42±1.14, 105.44±1.58; two-way ANOVA, p<0.0001; Figure 5D). Daily 0.3 mg/kg ALOS4 treatment significantly increased survival in comparison with saline-treated controls (Mantel-Cox, p<0.0001; Log-rank test for trend, p=0.0004; Figure 5E). Treatment of 16-week old non-inoculated C57BL/6 mice with daily i.p. injections of ALOS4 (0.3 mg/kg) for 28 days did not have any effect on animal weight (data not shown).

Thus ALOS4 demonstrated prominent anti-tumor effect *in vivo* against aggressive mouse melanoma. It inhibited tumor cell engraftment, slowed down the growth of established tumors and reduced the number of lung metastases.

## DISCUSSION

**Figure 6:**
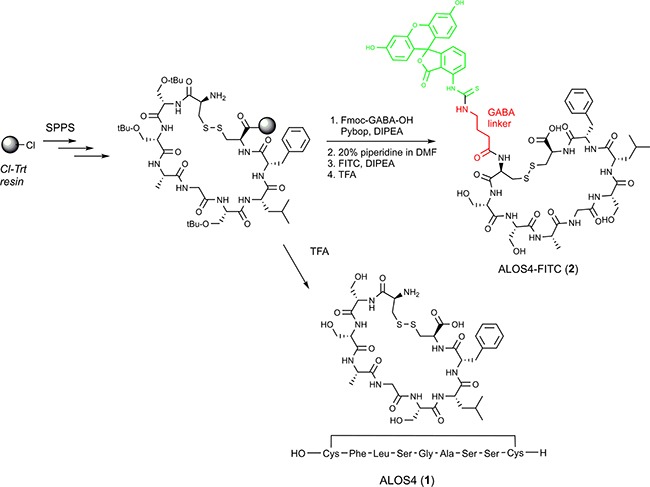
Synthesis of ALOS4(1) and ALOS4-FITC(2) Green structure represents FITC. Red structure represents the aminobutyric acid linker.

We report here a novel cyclic nonapeptide, ALOS4, with potent anticancer activity against an aggressive melanoma model. This integrin ligand does not resemble any other integrin antagonists [29]. We believe that the major action of ALOS4 is due to selective binding of the nonapeptide to the integrin αvβ3 due to the following observations: (1) the phage display revealed the pIII peptide was significantly enriched upon repeated panning with substrate adsorbed to integrin αvβ3; (2) saturation and heterologous binding studies further suggested ALOS4 specific binding affinity to αvβ3 (Figure 6).

Cyclic peptides can be restricted to a structure favorable for integrin binding because the presence of a disulfide bridge constrains its conformation and imposes structural stability [34, 35]. However, making the disulfide bridge complicates the peptide synthesis. Disulfide bonds usually form by air oxidation, but some peptides may need a treatment with an oxidant or dimethyl sulfoxide (DMSO). Fortunately, oxidation of phage library-derived synthetic peptides usually proceeds rapidly, because the peptides often adopt the energetically favorable conformation they have on the surface of the host cell for the phage [36]. From our peptide biopanning procedure and binding assay data, we postulate that a prime target for ALOS4 is the integrin αvβ3, however we do not discount the possibility of other binding sites within or outside of the integrin family.

Integrins perform critical functions in driving cancer progression and metastasis and represent attractive therapeutic targets [15, 37, 38]. Integrin αvβ3 in particular is overexpressed in many cancers: melanoma, prostate, pancreatic, breast, ovarian, cervical, glioblastoma (reviewed in [37]). A prominent binding and signaling site on αvβ3 recognizes ligands containing an Arg-Gly-Asp domain (RGD; [14, 25, 39]). Synthetic ligands for the RGD recognition site of αvβ3 have demonstrated *in vivo* anti-cancer activity, for example Cilengitide [40–42] and Abegrin [27]. This RGD motif is not present in ALOS4, suggesting possible alternative binding site on αvβ3.

Initial experiments demonstrated a lack of competition between ALOS4 and RGD-bearing ligands (data not shown). At least three additional non-RGD ligand binding sites on αvβ3 have been identified: thyroid hormone (T_3_/T_4_), resveratrol, and sex steroid sites (reviewed in [43]). Among these, the sex steroid site is known to be very proximal to the RGD recognition site as competition binding experiments have shown that RGD peptides inhibit dihydrotestosterone binding and associated cell proliferation [44]. Our results suggest that either ALOS4 acts on a non-RGD binding site in the integrin or simply displays a high integrin binding affinity and cannot be easily displaced, however further characterization is required.

Application of ALOS4 to mouse cancer models *in vivo* resulted in potent antitumor effects at relatively low doses of drug, without any overt toxicity. ALOS4 treatment of animals with subcutaneous melanoma resulted in significant inhibition of tumor growth and disease progression. In the metastatic model, survival of cancer cell-inoculated animals was significantly increased (Figure 5E). Animal weight gain was also preserved (Figure 5D). Lung nodule density, by gross anatomical inspection, was significantly reduced as well (Figure 5A-C). Experiments with ALOS4 on non-inoculated mice did not suggest any adverse effects, at least with respect to health effects relating to weight loss (data not shown).

The outcome of ALOS4 treatment on cell migration and cellular adhesion *in vitro* indicated that the peptide applies a slow, yet potent effect on cell activity related to metastatic cells. Indeed, integrins are acknowledged as implicitly important for facilitating cell migration in cancers [17, 45], as well as cancer cell interactions and cancer cell-platelet binding [16–18], the roles of which are critical for immune evasion and metastatic establishment of new tumor sites. Nodule formation was reduced in the i.v. inoculation mouse model of metastasis and tumor *establishment* was also reduced in the s.c. mouse model of melanoma. We suggest that these activities *in vitro* on interference with cellular mobility and attachment, possibly also cell-to-cell associations, likely underlie the observed anti-cancer effects in the animal models of melanoma studied here and describe the actions of an anti-metastatic agent.

In summary, we present here a novel cyclic peptide, ALOS4, with demonstrated anti-cancer effects in animal models. Further examination of the pharmacological targets of ALOS4, its effects in other cancer models and any potential side effects are currently being conducted. Additionally, its mechanism of action on proliferation, migration and invasion in additional tumor and non-tumor cells should be further examined. Elucidation of the binding site(s) recognized by ALOS4 using a radiolabeled analogue will allow for accurate pharmacological characterization and modeling of cellular effects, including possibly important secondary targets. Additional investigation of ALOS4 will reveal the underlying mechanisms of action and potentially lead to development of ALOS4 congeners with enhanced anti-cancer efficacy, leading to a promising new anti-cancer drug.

## MATERIALS AND METHODS

### Cell lines

B16F10 mouse melanoma cells were purchased from ATCC (cat# CRL-6475; Manassas, VA). All other reagents were purchased from Thermo-Fisher Scientific (Waltham, MA). B16F10 cells were cultured in DMEM+ medium (DMEM [cat# 41965-039], 10% fetal bovine serum [cat# 16000-036], 2% penicillin-streptomycin-glutamine [cat# 10378-016]). In migration assay experiments, B16F10 cells were cultured in RPMI+ medium (RPMI [cat# 11875-093], 10% fetal bovine serum [cat# 16000-036], 2 mM L-Gln [cat# 25030-149], 1 mM NaPyruvate [cat# 11360-070], 1% MEM non-essential amino acids [cat# 11140-050], 10 mM HEPES [cat# 15630-080], 1% penicillin-streptomycin-glutamine [cat# 10378-016], 50 μM 2-mercaptoethanol [cat# 21985-023]). ATCC employs a BacT/ALERT 3D sterility test as well as Hoechst DNA staining and direct culture inspection as anti-contamination quality control assays.

### Phage display

A M13 phage peptide surface display library containing 10^9^ peptides (cat# E8121L, Ph.D.™-C7C; New England Biolabs), was used for screening. The library contained phages displaying random disulfide-constrained nonapeptide sequences expressed at the amino-terminus of the M13 pIII minor coat protein.

Integrin αvβ3 was purchased from Millipore (cat# CC1018). Ninety-six well plates (cat# 655075, Greiner) were incubated overnight at 4°C with 200 μl/well of coating buffer (0.1 M NaHCO_3_, pH 8.6) containing 0.3 μg αvβ3. The phage library (2 × 10^11^pfu) was incubated with αvβ3-coated microplates (1 hour at RT). The whole procedure was repeated four times every time using library enriched in the previous cycle. All panning were performed according to the protocol (cat# E8121L, Ph.D.™-C7C; New England Biolabs). After the last round of biopanning, bound phages were eluted, amplified and randomly selected for further sequencing and bioinformatic analysis. The entire screening was run two times independently and peptides identified with the highest frequency in two screenings were selected for further analysis.

### Synthesis of ALOS4 and ALOS4-FITC

Synthesis of all the cyclic ALOS4 peptides was performed as described previously [46, 47]. Briefly, 2-chlorotrityl chloride resin (1.12 mmol/g) was placed in a reactor and suspended in DCM under nitrogen atmosphere. Then a mixture of Fmoc-Cys(Acm)-OH (2 eq) and DIPA (8 eq) in DCM was added. The resin loading reaction was allowed to proceed for 1-2 hr and then the resin was capped by an addition of a few drops of methanol (Figure 6). The Fmoc protecting group was removed with 20% piperidine/DMF (3 × 7 min) and then a linear SPPS was applied using standard Fmoc procedures introducing the amino acids (AA) in the following order: Fmoc-Gly-OH, Fmoc-Phe-OH, Fmoc-Leu-OH for 1a or Fmoc-Ser(tBu)-OH for 1b, Fmoc-Ala-OH. All the couplings were performed in DMF with 3-fold excess of AA and 6 eq of DIPA, using Pybop for activation. Each coupling cycle was conducted for 1-2 h. The completion of each coupling reaction and Fmoc removal were monitored by the ninhydrin test.

#### Cyclization step

After coupling of last AA, Fmoc-Cys(Acm)-OH and NMP wash, the resin was washed with 4:1 DMF/water (3 × 6.5 ml, 2 min each). A solution of I_2_ (10 eq, 35 mmol, 1.29 g) in 4:1 DMF/water (10 ml) was added, followed by agitation at RT for 1 hr to afford the disulfide bridge cyclization. The peptidyl – resin was filtered and washed extensively with 4:1 DMF/water (7 × 10 ml, 2 min each), DMF (6 × 10 ml, 2 min each), DCM (6 × 10 ml, 2 min each), CHCl_3_ (4 × 10 ml, 2 min each), 2% ascorbic acid in DMF (6 × 10 ml, 2 min each), and DMF (6 × 10 ml, 2 min each). At this point the resin was divided in two: first portion underwent cleavage to give ALOS4 and the second portion was used for coupling of FITC through GABA linker (ALOS4-FITC). The cleavage procedure is mentioned below

#### Coupling of Fmoc-γ-aminobutyric acid (linker)

Fmoc-γ-aminobutyric acid (3 eq, 10.5 mmol, 0.49 g) dissolved in NMP (7 ml) was activated with PyBroP (3 eq) and DIEA (6 eq) for 4 min at RT, and then was transferred to the reaction vessel and allowed to react for 1h at RT. After post coupling wash and Fmoc-deprotection (20% piperidine in NMP (10 ml) for 15 min), the second portion of peptidyl resin was ready for coupling with fluorescein isothiocyanate (FITC).

#### Coupling of fluorescein isothiocyanate (FITC)

Fluorescein isothiocyanate (3 eq,) was dissolved in 50 ml NMP and added to the peptidyl – resin. After 1h the resin was washed with NMP (7 times, 7 ml, 2 min each time). Completion of reaction was monitored by ninhydrin test (Kaiser test – result yellow).

#### Cleavage of the peptide from the resin

Resin portions were washed with NMP and the resin washed with DCM (4 times, 7 ml, 2 min each time), MeOH (4 times, 7 ml, 2 min each time), DCM (4 times, 7 ml, 2 min each time) and dried under vacuum for 20 min. The peptide was cleaved from the resin using cocktail solution of 95:2.5:2.5 TFA/TIS/H_2_O (9 ml) for 5 min at 0°C under argon and then 1 h at RT under argon. The resin was filtered and washed with the cocktail (1 ml) and TFA (1.5 ml). The filtrate solution was evaporated to give an oily residue. Upon the addition of cold Et_2_O, the oily residue solidified. Centrifugation and decantation of the Et_2_O layer and repeated treatment with additional cold Et_2_O afforded the crude peptidyl residue which was purified by RP-HPLC and kept in dark at -20°C under argon.

Peptides were purified after synthesis by reverse-phase HPLC (C18) and their structures confirmed by mass spectroscopy. For ALOS4(1): yellowish solid, (89% yield) LC-MS: RT = 7.60 min; HRMS: ESI-MS m/z calcd: 871.3193, found: 872.3272 (MH^+^), calcd: 894.3083, found: 894.3097 (MNa^+^); For ALOS4-FITC(2): orange solid, (68% yield) HRMS: ESI-MS m/z calcd: 1345.4065, found: 673.8039 (M2H^++^/2), calcd: 1369.4132, found: 684.7078 (MHNa^++^/2). Peptides were dissolved in saline containing 0.02% BSA, aliquoted and stored at -80°C until use.

### Binding assay

Integrin αvβ3 was purchased from Millipore (cat# CC1018). Ninety-six well plates (cat# 655075, Greiner) were incubated overnight at 4°C with 200 μl/well of TBST coating buffer (pH 7.6) containing 0.3 μg αvβ3. Wells were subsequently washed twice with TBST buffer and blocked for two hours with 5% nonfat dry milk in TBS. Plates were then washed four times with 200 μl TBST.

For saturation assays, integrin-coated plates were incubated with FITC-labeled peptide solutions (0.003-100 μM) in PBS containing 0.02% BSA for 30 min at RT. For competition assays, plates were incubated with different concentrations of unlabeled peptides for 30 min in RT followed by addition of fixed concentrations of FITC-labeled peptides and an additional incubation for 30 min. After incubation wells were washed twice with TBST. After second wash, 100 μl of TBST was added to each well and fluorescence signal was read at excitation of 490 nm and emission 525 nm.

### Cell viability assays

B16F10 cells were plated in 96 well plates (cat# 655075, Greiner) at 1×10^3^ per well. The following day, cells were treated in triplicate serial dilutions of ALOS4 (0.003-100 μM), 9-aminoacridine (50 μM, positive control; cat# 92817, Sigma-Aldrich) or drug-free medium (negative control) and incubated for 48 or 72 hours. Cell viability then was assessed via resazurin (cat# R7017, Sigma-Aldrich) staining. Resazurin (150 μM, 0.2 μm filter-sterilized in Dulbecco's PBS; pH 7.4) was added to cell cultures and incubated 4 hours (37°C, 5% CO_2_). The resorufin product was measured fluorometrically (560 nm excitation/590 nm emission).

### Cell migration assays

B16F10 cells were grown until approximately 90% confluence and subsequently treated with ALOS4 (0.03-3 μM) each 24 h for 24, 48, 60 or 72 h. Cells were scratched with a 200 μl tip, washed twice with serum-free media and replaced with reduced-serum media (0.5%). Wells were imaged using inverted microscopy and re-imaged at the same coordinates after 8 h incubation. Relative closure of gap was evaluated using TScratch image analysis software to determine migration rate of cells [48].

### Cell adhesion assay

B16F10 cells treated with concentrations of ALOS4 (0.03-3 μM) were cultured (37°C, 5% CO_2_) at cell concentrations preventing confluence by end of assay pretreatment interval (24 or 48 hours treatment). Cultures were washed twice with serum-free media, trypsinized to microcentrifuge tubes and precipitated (1500×g, 10 min). Cells were resuspended, cell numbers were assessed by manual counting with a hemocytometer and plated in media at 8×10^5^ cells per well. Concentrations of ALOS4 identical to pretreatment stage were added and cultures were allowed to incubate for an additional 24 hours (37°C, 5% CO_2_). At assay endpoint, cultures were washed twice with serum-free media, trypsinized to microcentrifuge tubes and precipitated (1500×g, 10 min). Cells were resuspended and cell numbers were assessed by manual counting with a hemocytometer.

### Immunofluorescence microscopy

Poly-L-lysine (1 h, 37°C; cat# P8920, Sigma-Aldrich) coated slides (cat# PEZGS0816, Merck) were washed 2x with PBS (pH 7.4), 1.5×10^4^ B16F10 cells per well were seeded in RPMI (10% FCS) and allowed to incubate overnight (37°C, 5% CO_2_). ALOS4-FITC (10 μM) was added to slide wells up to 60 min (37°C, 5% CO_2_), then cells were fixed in paraformaldehyde (4%, 10 min). Following PBS washes (2x) and removal of excess buffer, cells were stained with a mounting medium containing DAPI (cat# H-1500, Vector Laboratories, Burlingame, CA). Slides were imaged by confocal microscopy.

### Animal experiments

Ethics Statement: All animal research was conducted in accordance with guidelines set by Israel National Ministry of Health and was supervised by the Institutional Animal Care and Use Committee of Ariel University.

Animal experiments were performed on C57BL/6 mice (Harlan, Israel). The mice were housed in a standard animal laboratory with access to water and food *ad libitum*. They were kept under constant environmental conditions with a 12-hour light-dark cycle.

### Mouse model of metastatic melanoma

B16F10 cells were resuspended in 0.9% saline and maintained at 4°C before inoculation. Mice (16-week old) were inoculated with 5×10^4^ cells in 100 μl of saline by tail vein injection using a 1 ml syringe with a 27G needle. Mice were randomly divided into groups of animals and injected with saline or ALOS4 (0.1, 0.3 or 0.5 mg/kg) i.p. daily beginning one day after inoculation until the end of experiment. Mice were monitored daily and weighed at least three times a week. For the analysis of peptide effects on melanoma lung metastasis mice were euthanized 18 days after inoculation. In survival experiments, animals were monitored until appearance of moribund signs such as lethargy, 20% or more weight loss, hunched position and epilepsy. Moribund mice were euthanized with CO_2_ anesthesia followed by cervical dislocation. Organs were dissected and assessed by gross anatomical inspection. The lungs were rinsed in saline and fixed with Bouin's solution. The total number of visible nodules on the lung surface per animal was counted.

### Mouse model of subcutaneous melanoma

C57BL/6 mice (16-week old) were inoculated with B16F10 melanoma cells (5×10^4^/mouse s.c. in 200 μl of PBS) into the right lateral side of the pelvis. Following inoculation, B16F10 cells formed a palpable tumor of which the mean group tumor size was approximately 100 mm^3^ by day 13 post-inoculation. Mice were randomized into groups and treated with saline or ALOS4 (0.1, 0.3 or 0.5 mg/kg) i.p. daily beginning post-inoculation day 13. Tumor volume was measured using digital caliper and calculated using formula (π/6 × width × length × height) and survival was monitored until the tumor volume reached 200 mm^3^.

### Statistical analysis

ALOS4-FITC binding assay data was analyzed by single-site binding non-linear regression [Y = Bmax × X/(Kd + X] after correction for non-specific binding using a ALOS4-FITC standard. ALOS4-FITC/ALOS4 displacement assay was analyzed by log agonist versus response non-linear regression [Y = Minimum + (Maximum-Minimum)/(1+10^((LogEC50-X) × Hill Slope))^] with a constrained Hill Slope of 1.0. Two-way ANOVA with Bonferroni means separation test was used for multiple comparisons and single comparisons were performed by unpaired t-test (significance level p<0.05). Survival data was analyzed by Mantel-Cox statistic followed by a log-rank test for dose-dependent trend. All data expressed as mean ± SEM.

## References

[1] Kampen KR (2011). Membrane proteins: the key players of a cancer cell. The Journal of membrane biology.

[2] Hynes RO (2002). Integrins: bidirectional, allosteric signaling machines. Cell.

[3] Goodman SL, Picard M (2012). Integrins as therapeutic targets. Trends in pharmacological sciences.

[4] Moore SF, Hunter RW, Harper MT, Savage JS, Siddiq S, Westbury SK, Poole AW, Mumford AD, Hers I (2013). Dysfunction of the PI3 kinase/Rap1/integrin alpha(IIb)beta(3) pathway underlies ex vivo platelet hypoactivity in essential thrombocythemia. Blood.

[5] Handa M, Watanabe K, Kawai Y, Kamata T, Koyama T, Nagai H, Ikeda Y (1995). Platelet unresponsiveness to collagen: involvement of glycoprotein Ia-IIa (alpha 2 beta 1 integrin) deficiency associated with a myeloproliferative disorder. Thrombosis and haemostasis.

[6] Sayedyahossein S, Dagnino L (2013). Integrins and small GTPases as modulators of phagocytosis. International review of cell and molecular biology.

[7] Worthington JJ, Fenton TM, Czajkowska BI, Klementowicz JE, Travis MA (2012). Regulation of TGFbeta in the immune system: an emerging role for integrins and dendritic cells. Immunobiology.

[8] Worthington JJ, Klementowicz JE, Travis MA (2011). TGFbeta: a sleeping giant awoken by integrins. Trends in biochemical sciences.

[9] Koivisto L, Heino J, Hakkinen L, Larjava H (2014). Integrins in Wound Healing. Advances in wound care.

[10] Mitroulis I, Alexaki VI, Kourtzelis I, Ziogas A, Hajishengallis G, Chavakis T (2015). Leukocyte integrins: role in leukocyte recruitment and as therapeutic targets in inflammatory disease. Pharmacology & therapeutics.

[11] Cicala C, Arthos J, Fauci AS (2011). HIV-1 envelope, integrins and co-receptor use in mucosal transmission of HIV. Journal of translational medicine.

[12] Rozdzinski E, Tuomanen E (1995). Adhesion of microbial pathogens to leukocyte integrins: methods to study ligand mimicry. Methods in enzymology.

[13] Rozdzinski E, Tuomanen E (1994). Interactions of bacteria with leukocyte integrins. Methods in enzymology.

[14] Takada Y, Ye X, Simon S (2007). The integrins. Genome Biol.

[15] Kumar CC (2003). Integrin alpha v beta 3 as a therapeutic target for blocking tumor-induced angiogenesis. Current drug targets.

[16] Felding-Habermann B, Habermann R, Saldivar E, Ruggeri ZM (1996). Role of beta3 integrins in melanoma cell adhesion to activated platelets under flow. The Journal of biological chemistry.

[17] Felding-Habermann B, O'Toole TE, Smith JW, Fransvea E, Ruggeri ZM, Ginsberg MH, Hughes PE, Pampori N, Shattil SJ, Saven A, Mueller BM (2001). Integrin activation controls metastasis in human breast cancer. Proceedings of the National Academy of Sciences of the United States of America.

[18] Weber MR, Zuka M, Lorger M, Tschan M, Torbett BE, Zijlstra A, Quigley JP, Staflin K, Eliceiri BP, Krueger JS, Marchese P, Ruggeri ZM, Felding BH (2016). Activated tumor cell integrin alphavbeta3 cooperates with platelets to promote extravasation and metastasis from the blood stream. Thrombosis research.

[19] Seftor RE, Seftor EA, Gehlsen KR, Stetler-Stevenson WG, Brown PD, Ruoslahti E, Hendrix MJ (1992). Role of the alpha v beta 3 integrin in human melanoma cell invasion. Proceedings of the National Academy of Sciences of the United States of America.

[20] Bello L, Francolini M, Marthyn P, Zhang J, Carroll RS, Nikas DC, Strasser JF, Villani R, Cheresh DA, Black PM (2001). Alpha(v)beta3 and alpha(v)beta5 integrin expression in glioma periphery. Neurosurgery.

[21] McCabe NP, De S, Vasanji A, Brainard J, Byzova TV (2007). Prostate cancer specific integrin alphavbeta3 modulates bone metastatic growth and tissue remodeling. Oncogene.

[22] van den Hoogen C, van der Horst G, Cheung H, Buijs JT, Pelger RC, van der Pluijm G (2011). Integrin alphav expression is required for the acquisition of a metastatic stem/progenitor cell phenotype in human prostate cancer. The American journal of pathology.

[23] van der Horst G, van den Hoogen C, Buijs JT, Cheung H, Bloys H, Pelger RC, Lorenzon G, Heckmann B, Feyen J, Pujuguet P, Blanque R, Clement-Lacroix P, van der Pluijm G (2011). Targeting of alpha(v)-integrins in stem/progenitor cells and supportive microenvironment impairs bone metastasis in human prostate cancer. Neoplasia.

[24] Takayama S, Ishii S, Ikeda T, Masamura S, Doi M, Kitajima M (2005). The relationship between bone metastasis from human breast cancer and integrin alpha(v)beta3 expression. Anticancer research.

[25] D'souza SE, Ginsberg MH, Plow EF (1991). Arginyl-glycyl-aspartic acid (RGD): a cell adhesion motif. Trends in biochemical sciences.

[26] Carter A (2010). Integrins as target: first phase III trial launches, but questions remain. J Natl Cancer Inst.

[27] Hersey P, Sosman J, O'Day S, Richards J, Bedikian A, Gonzalez R, Sharfman W, Weber R, Logan T, Buzoianu M, Hammershaimb L, Kirkwood JM, Etaracizumab Melanoma Study G (2010). A randomized phase 2 study of etaracizumab, a monoclonal antibody against integrin alpha(v)beta(3), + or - dacarbazine in patients with stage IV metastatic melanoma. Cancer.

[28] Rodgers UR, Weiss AS (2004). Integrin alpha v beta 3 binds a unique non-RGD site near the C-terminus of human tropoelastin. Biochimie.

[29] Kapp TG, Rechenmacher F, Sobahi TR, Kessler H (2013). Integrin modulators: a patent review. Expert opinion on therapeutic patents.

[30] Seftor RE (1998). Role of the beta3 integrin subunit in human primary melanoma progression: multifunctional activities associated with alpha(v)beta3 integrin expression. The American journal of pathology.

[31] Watson-Hurst K, Becker D (2006). The role of N-cadherin, MCAM and beta3 integrin in melanoma progression, proliferation, migration and invasion. Cancer biology & therapy.

[32] Hofmann UB, Westphal JR, Waas ET, Becker JC, Ruiter DJ, van Muijen GN (2000). Coexpression of integrin alpha(v)beta3 and matrix metalloproteinase-2 (MMP-2) coincides with MMP-2 activation: correlation with melanoma progression. The Journal of investigative dermatology.

[33] Petitclerc E, Stromblad S, von Schalscha TL, Mitjans F, Piulats J, Montgomery AM, Cheresh DA, Brooks PC (1999). Integrin alpha(v)beta3 promotes M21 melanoma growth in human skin by regulating tumor cell survival. Cancer research.

[34] Mann M, Jensen ON (2003). Proteomic analysis of post-translational modifications. Nature biotechnology.

[35] Seo J, Lee KJ (2004). Post-translational modifications and their biological functions: proteomic analysis and systematic approaches. Journal of biochemistry and molecular biology.

[36] O'Neil KT, Hoess RH, Jackson SA, Ramachandran NS, Mousa SA, DeGrado WF (1992). Identification of novel peptide antagonists for GPIIb/IIIa from a conformationally constrained phage peptide library. Proteins.

[37] Desgrosellier JS, Cheresh DA (2010). Integrins in cancer: biological implications and therapeutic opportunities. Nature reviews Cancer.

[38] Liu Z, Wang F, Chen X (2008). Integrin alpha(v)beta(3)-Targeted Cancer Therapy. Drug development research.

[39] Ruoslahti E (1996). RGD and other recognition sequences for integrins. Annual review of cell and developmental biology.

[40] Stupp R, Hegi ME, Gorlia T, Erridge SC, Perry J, Hong YK, Aldape KD, Lhermitte B, Pietsch T, Grujicic D, Steinbach JP, Wick W, Tarnawski R, Nam DH, Hau P, Weyerbrock A (2014). Cilengitide combined with standard treatment for patients with newly diagnosed glioblastoma with methylated MGMT promoter (CENTRIC EORTC 26071-22072 study): a multicentre, randomised, open-label, phase 3 trial. The Lancet Oncology.

[41] Stupp R, Hegi ME, Neyns B, Goldbrunner R, Schlegel U, Clement PM, Grabenbauer GG, Ochsenbein AF, Simon M, Dietrich PY, Pietsch T, Hicking C, Tonn JC, Diserens AC, Pica A, Hermisson M (2010). Phase I/IIa study of cilengitide and temozolomide with concomitant radiotherapy followed by cilengitide and temozolomide maintenance therapy in patients with newly diagnosed glioblastoma. Journal of clinical oncology.

[42] Reardon DA, Fink KL, Mikkelsen T, Cloughesy TF, O'Neill A, Plotkin S, Glantz M, Ravin P, Raizer JJ, Rich KM, Schiff D, Shapiro WR, Burdette-Radoux S, Dropcho EJ, Wittemer SM, Nippgen J (2008). Randomized phase II study of cilengitide, an integrin-targeting arginine-glycine-aspartic acid peptide, in recurrent glioblastoma multiforme. Journal of clinical oncology.

[43] Davis PJ, Mousa SA, Cody V, Tang HY, Lin HY (2013). Small molecule hormone or hormone-like ligands of integrin alphaVbeta3: implications for cancer cell behavior. Hormones & cancer.

[44] Lin HY, Sun M, Lin C, Tang HY, London D, Shih A, Davis FB, Davis PJ (2009). Androgen-induced human breast cancer cell proliferation is mediated by discrete mechanisms in estrogen receptor-alpha-positive and -negative breast cancer cells. The Journal of steroid biochemistry and molecular biology.

[45] Huttenlocher A, Horwitz AR (2011). Integrins in cell migration. Cold Spring Harbor perspectives in biology.

[46] Gilad Y, Firer MA, Rozovsky A, Ragozin E, Redko B, Albeck A, Gellerman G (2014). “Switch off/switch on” regulation of drug cytotoxicity by conjugation to a cell targeting peptide. European journal of medicinal chemistry.

[47] Redko B, Ragozin E, Bazylevich A, Tuchinsky H, Albeck A, Shekhter Zahavi T, Oron-Herman M, Kostenich G, Gellerman G (2015). Synthesis, drug release and biological evaluation of new anticancer drug-bioconjugates containing somatostatin backbone cyclic analog as a targeting moiety. Biopolymers.

[48] Geback T, Schulz MM, Koumoutsakos P, Detmar M (2009). TScratch: a novel and simple software tool for automated analysis of monolayer wound healing assays. BioTechniques.

